# Effects of Hearing Loss and Cognitive Load on Speech Recognition with Competing Talkers

**DOI:** 10.3389/fpsyg.2016.00301

**Published:** 2016-03-04

**Authors:** Hartmut Meister, Stefan Schreitmüller, Magdalene Ortmann, Sebastian Rählmann, Martin Walger

**Affiliations:** ^1^Jean-Uhrmacher-Institute for Clinical ENT-Research, University of CologneCologne, Germany; ^2^Clinic of Otorhinolaryngology, Head and Neck Surgery, University of CologneCologne, Germany

**Keywords:** speech recognition, competing talkers, attention, working memory, age-related hearing loss

## Abstract

Everyday communication frequently comprises situations with more than one talker speaking at a time. These situations are challenging since they pose high attentional and memory demands placing cognitive load on the listener. Hearing impairment additionally exacerbates communication problems under these circumstances. We examined the effects of hearing loss and attention tasks on speech recognition with competing talkers in older adults with and without hearing impairment. We hypothesized that hearing loss would affect word identification, talker separation and word recall and that the difficulties experienced by the hearing impaired listeners would be especially pronounced in a task with high attentional and memory demands. Two listener groups closely matched for their age and neuropsychological profile but differing in hearing acuity were examined regarding their speech recognition with competing talkers in two different tasks. One task required repeating back words from one target talker (1TT) while ignoring the competing talker whereas the other required repeating back words from both talkers (2TT). The competing talkers differed with respect to their voice characteristics. Moreover, sentences either with low or high context were used in order to consider linguistic properties. Compared to their normal hearing peers, listeners with hearing loss revealed limited speech recognition in both tasks. Their difficulties were especially pronounced in the more demanding 2TT task. In order to shed light on the underlying mechanisms, different error sources, namely having misunderstood, confused, or omitted words were investigated. Misunderstanding and omitting words were more frequently observed in the hearing impaired than in the normal hearing listeners. In line with common speech perception models, it is suggested that these effects are related to impaired object formation and taxed working memory capacity (WMC). In a *post-hoc* analysis, the listeners were further separated with respect to their WMC. It appeared that higher capacity could be used in the sense of a compensatory mechanism with respect to the adverse effects of hearing loss, especially with low context speech.

## Introduction

Age-related hearing loss is a common chronic condition in older persons (Zhan et al., 2010; Lin et al., 2011). It causes communication problems, especially in demanding listening situations, such as when speech is masked with noise or when competing talkers are present (Festen and Plomp, 1990; Kiessling et al., 2003; Summers and Molis, 2004). There is a growing body of evidence suggesting that cognitive factors play an important role in these situations (Akeroyd, 2008; Humes, 2013). A number of studies have shown a relationship between speech recognition and working memory capacity (WMC). Working memory refers to short-term maintenance and processing of information supporting ongoing and upcoming actions (e.g., Baddeley, 2010; Eriksson et al., 2015; Mansouri et al., 2015). It is characterized by a limited capacity system typically declining with age (e.g., Nyberg et al., 2012).

Another basic cognitive factor involved in speech understanding is attention (e.g., Bronkhorst, 2015). In a multitalker environment, attention refers to the ability to selectively focus on a target talker while inhibiting competing information, or to divide attention to or switch between different talkers (McDowd, 2007). Though frequently used as autonomous definitions, working memory and attention are substantially intertwined (Barrouillet et al., 2004; Engle and Kane, 2004) and both attributed to the concept of core executive functions (e.g., Diamond, 2013).

Both attention and working memory are reflected in common models of speech understanding in adverse listening situations. The concept of auditory scene analysis (Bregman, 1990) assumes that in a multitalker environment, at first, auditory objects are established (Griffiths and Warren, 2004; Shinn-Cunningham and Best, 2008). After object formation, the auditory objects are grouped into auditory streams. Different acoustic cues, such as the talker's fundamental frequency or other voice characteristics such as formant frequencies, are used for stream build up (Shinn-Cunningham and Best, 2008; Moore and Gockel, 2012). Following this concept, attention can then be selectively directed to the talker of interest while inhibiting irrelevant information, or it can be redirected to another auditory stream.

Sörqvist (2010) and Rönnberg et al. (2013) describe that inhibition of irrelevant information or dividing attention between different sources are associated with individual WMC of the listener. In the framework of their “ease of language understanding” (ELU) model (Rönnberg, 2003; Rönnberg et al., 2008, 2013), they describe different memory domains associated with the processing of speech. Basically, the ELU model postulates that multimodal (i.e., auditory, visual) speech information is bound into a phonological representation in an episodic buffer based on a continous process that feeds forward syllables in rapid succession. Entries of this buffer are matched with corresponding representations in semantic long-term memory (LTM). Under ideal circumstances, this *implicit* process allows rapid and automatic lexical retrieval. However, if the speech input is altered—for example, due to hearing loss, masking, artifacts of signal processing, etc.—it might not be precise enough to match the representations in semantic LTM. The model then assumes that *explicit* cognitive processes come into play to compensate for the mismatch: The altered information has to be stored and further processed, engaging short-term and working memory, respectively. This process might include inference-making, semantic integration, switching of attention, storing of information, and inhibiting irrelevant information (Rönnberg et al., 2013). Following the ELU model, WMC is essential for executing these explicit processes in order to overcome the disruption of the automatic implicit process. In conjunction with this, the ELU model also considers lexical context as an important factor aiding speech recognition. The use of context relies on linguistic knowledge and narrows down the set of lexical candidates in the speech stream accordingly supporting explicit cognitive processing (Rönnberg et al., 2013). Linguistic knowledge and the rules for its use are preserved in older age and thus might be used to counteract effects of cognitive decline and hearing impairment associated with aging (e.g., Wingfield et al., 2015).

Against the background of these model considerations, the present study attempted to examine mechanisms in older adults with respect to speech recognition when competing talkers are present. Concretely, we were interested in the effects of hearing impairment and attention tasks differing in cognitive load. Therefore, older persons with typical age-related hearing loss and a matched control group of older persons with clinically normal hearing thresholds were requested to repeat back words either from a single target talker or from two target talkers in a competing talker paradigm. Thus, tasks differed regarding their attentional and memory demands. We further examined the effects of context with these two tasks by presenting concurrent speech streams with lower and higher word predictability. Three different error sources reflecting word object formation, stream segregation and word recall were determined in order to shed light on the question of at which stage of the processing problems occur for the listeners. It was hypothesized that the hearing-impaired individuals exhibit significantly greater speech recognition problems than their normal-hearing peers at all processing stages reflecting in degraded object formation, stream segregation, and word recall. We anticipated that the difficulties of the HI listeners were especially pronounced under higher cognitive load. We further hypothesized that both listener groups make use of context to promote speech recognition with competing talkers.

## Methods

### Speech materials

Two commonly used German speech audiometric test materials were administered, namely the Oldenburg sentence test (“OLSA,” Wagener et al., 1999) and the Göttingen sentence test (“GOESA,” Kollmeier and Wesselkamp, 1997). The OLSA presents low context speech with a fixed five-word syntactic structure (name–verb–numeral–adjective–object, such as “Stefan kauft sieben nasse Schuhe”/“Stefan buys seven wet shoes”). These sentences are syntactically correct but semantically unpredictable. Using the j-factor model (Boothroyd and Nittrouer, 1988), calculating a measure for the predictability of the OLSA corpus yields a value of *j* = 4.3 (i.e., an average of 4.3 parts of the sentences are statistically independent). The GOESA presents high context speech and includes everyday sentences with three- to seven-word lengths and with a high word predictability of *j* = 2.5 (Bronkhorst et al., 2002). Only the five-word sentences from GOESA (such as “Adler fliegen tausend Meter hoch”/“eagles fly thousand meters high”) were used in order to match the length of the OLSA sentences. The same male speaker produced both GOESA and OLSA materials.

In order to provide distinct acoustic cues for the separation of target and masker, the sentences were modified with respect to the fundamental frequency (F0) and formant frequencies using “praat” (Boersma and Weenink, 2001). F0 of the original utterances was shifted by +80 Hz and formant frequencies were shifted by +16%, thereby yielding the characteristics of a female talker (Darwin et al., 2003). Original and modified sentences thus differed solely in these characteristics, with all other attributes (such as prosody, speaking rate, etc.) being identical. Acoustic modifications yielded naturally sounding stimuli and the participants were not aware of the female voice being an artificial adaptation of the male talker.

Stimuli were generated by superimposing two sentences, one with the male voice and one with the female voice. The corresponding sentences were identical in duration. The level of the sentences was not modified thus yielding a mean target-masker ratio (TMR) of 0 dB across all sentence pairs. In order to consider not only acoustic characteristics of different talkers, but also linguistic properties, the superimposed sentences were either drawn from the low context speech material of the OLSA stimulus type (denoted as LC/LC, where LC stands for low context) or from both low context (OLSA) and high context sentences of the GOESA (denoted as LC/HC). In the latter case, both sentence sets were used as a target as well as a masker, depending on the given voice characteristics (speaker gender as the target cue, see procedures). The number of OLSA and GOESA targets was balanced. Stimuli were presented at an average level of 70 dB SPL via a free-field loudspeaker placed in front of the participant's head at a distance of 1.2 m in a sound-treated booth.

### Procedures

Procedures based on methods described by Humes et al. (2006) and Meister et al. (2013). Speech recognition was assessed during two different attention tasks. With the “one target talker” task (1TT), the participants were requested to selectively attend to a target talker and to repeat back as many words as possible from the target sentences while ignoring the competing masker sentences. Prior to each stimulus, the target sentence was indicated by requesting the participant to listen to either the female or the male voice. This information was updated from trial to trial with a balanced proportion of male and female targets. With the more demanding “two target talkers” task (2TT) the participants were requested to repeat back as many words as possible from both talkers and to correctly assign them to the male and the female voice. Thus, both tasks differed with regard to their attentional and memory requirements whereas perceptual load was identical due to the use of identical stimuli. With both tasks the listeners were encouraged to guess in case they were uncertain about the words presented. Measurements were performed with three test lists with 14 stimuli per condition, yielding 168 presentations in total (42 stimuli [3 lists with 14 sentence pairs each] × 2 target talker tasks [1TT, 2TT] × 2 stimulus types [LC/LC, LC/HC]). To avoid order effects the order of tasks and stimulus types was randomized and the lists were randomly assigned to the different conditions.

Prior to the measurements, the participants were intensively familiarized with the stimulus materials and the procedures. Stimuli presented during familiarization were discarded for the measurements.

### Participants

Fourteen older adults aged 58–79 years (mean 68.3 years) with good hearing (denoted as “normal hearing” (NH) listeners in the following) and 14 older adults with typical age related hearing loss (denoted as hearing impaired (HI) listeners) aged 60–85 years (mean 69.6 years) participated in the study. Hearing loss was predominantly symmetrical, with between-ear differences typically less than 15 dB HL. None of the listeners was provided with hearing aids. Mean pure-tone thresholds are given in Table 1.

**Table 1:** **Better ear hearing loss (BEHL) of the normal-hearing (NH) and hearing-impaired (HI) listeners for the frequencies 0.125–8 kHz**.

**f (kHz)**	**0.125**	**0.25**	**0.5**	**1**	**2**	**4**	**6**	**8**
NH (dB HL)	11.8±6.4	9.6±6.9	11.8±4.5	7.5±3.7	13.2±7.5	11.8±7.2	12.9±9.9	24.3±8.4
HI (dB HL)	15.7±7.8	16.1±9.9	19.6±10.9	21.4±9.5	37.1±12.1	50.4±14.9	62.1±17.6	71.1±19.5

Mean and standard deviation are given.

Both groups underwent cognitive screening using the DemTect inventory (Kalbe et al., 2004). All participants passed the cognitive screening (score > 12 in the DemTect). In order to match the two groups with regard to their neuropsychological profile a test battery addressing different cognitive domains was administered. These tests tapped into attention and concentration (test d2, Brickenkamp, 1962), attention and task switching (Trailmaking test, Reitan, 1958), reasoning and fluid intelligence (Leistungsprüfsystem LPS-4, Horn, 1983), crystallized intelligence (Mehrfachwortschatztest MWT-B, Lehrl, 2005) as well as WMC (Verbaler Lern- und Merkfähigkeitstest VLMT, Helmstädter et al., 2001). The VLMT was further used for a *post-hoc* grouping criterion (i.e., median split) of the listeners. With the VLMT lists of 15 words were visually presented and the participants were requested to recall back as many words as possible. This procedure was repeated five times and the mean across the repetitions was calculated as the outcome value. Thus, a value of 10 corresponds to 10/15 words recalled per list in average. The test primarily addresses verbal short-term memory and learning abilities, but also captures the individual WMC of the participant (see Elger et al., 1997; Helmstädter et al., 2001; Van der Elst et al., 2005 for the English version of the VLMT). Group results of the VLMT are shown in Table 2. Importantly, there were no significant group differences with all neuropsychological measures assessed, namely the test d2, the Trailmaking test, the LPS-4, the MWT-B and the VLMT (independent samples *t*-tests, all *p* > 0.05).

**Table 2:** **VLMT scores for the listener groups and the post-hoc median split of the VLMT**.

	**VLMT score**	***Post-hoc median split***	**VLMT score**
NH	10.3±1.2	below (*n* = 7)	9.4±0.8
		above (*n* = 7)	11.2±0.7
HI	10.2±1.7	below (*n* = 7)	8.7±0.7
		above (*n* = 7)	11.6±1.2

Mean and standard deviation are given.

All participants provided their written informed consent prior to the experiments. The study was approved by the ethics committee of the University of Cologne.

### Analyses

Following the methods described by Meister et al. (2013), the participants' responses with the speech recognition tests were audio recorded in order to allow for a detailed analysis of errors. Three types of errors were documented, namely substitutions, confusions and omissions. Substitutions were indicated if a word repeated back did not match the word presented. These words were predominantly lexical neighbors, that is, at least one phonological element of a word was correct but other parts were misunderstood (such as taking “Dosen” for “Rosen”). Confusions were indicated if overlapping words from the target and masker talker were mixed up, that is when a word uttered by the female voice was spuriously assigned to the male voice and vice versa. Due to the regular syntactic structure of all sentences, word positions were not confused. Omissions were indicated if a word presented was not repeated back.

Mixed design ANOVAs for the number of words repeated back and the number of different errors were conducted, with task (1TT, 2TT) and stimulus type (LC/LC, LC/HC) as within-subject variables, and listener group (NH, HI) as the between-subject variable. Moreover, for a *post-hoc* examination of the influence of WMC on speech recognition, a median split was performed based on the VLMT scores (above median: VLMT↑, below median: VLMT↓), and used as a further between-subject variable. Log-transforms were applied since not all data were normally distributed. All statistical analyses were performed using IBM SPSS Statistics 22.

## Results

**Figure 1:**
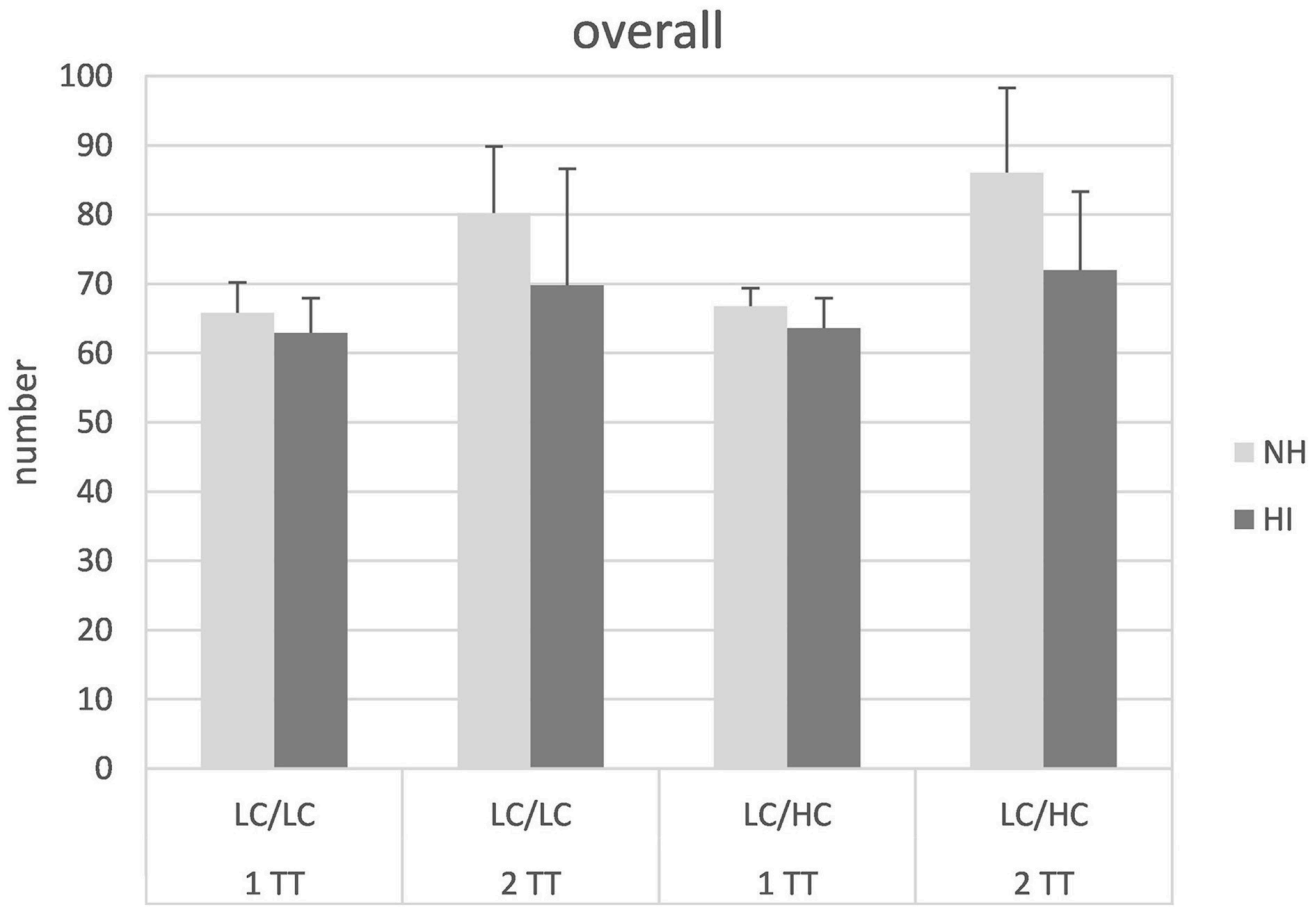
**Overall number of words repeated back in normal-hearing (NH) and hearing-impaired (HI) listeners for the different tasks and stimulus types**. LC, low context; HC, high context; 1 TT, one target talker, 2 TT, two target talkers. Mean across one test list and standard deviation are given.

Figure 1 shows the *overall* number of words repeated back, irrespective of substitution or confusion errors. The outcome is given as the average across the three tests lists (14 sentence pairs each) presented for each condition (i.e., maximum 70 words in the 1TT and 140 words in the 2TT task). In general, the 2TT task obviously yielded more words repeated back than the 1TT task and the NH listeners repeated back more words than the HI listeners. Subjecting the data to a mixed design ANOVA revealed significant main effects of task [*F*_(1, 26)_ = 27.28, *p* < 0.001] and group [*F*_(1, 26)_ = 8.24, *p* = 0.008]. Moreover, a significant interaction task × group [*F*_(1, 26)_ = 4.96, *p* = 0.035] could be observed. This significant interaction was evaluated further. *Post-hoc* independent samples *t*-tests revealed that the difference between the 1TT and the 2TT task was significantly greater in the NH listeners than in the HI listeners [*t*_(1, 54)_ = 2.58, *p* = 0.012]. No other main effects or interactions were significant.

**Figure 2:**
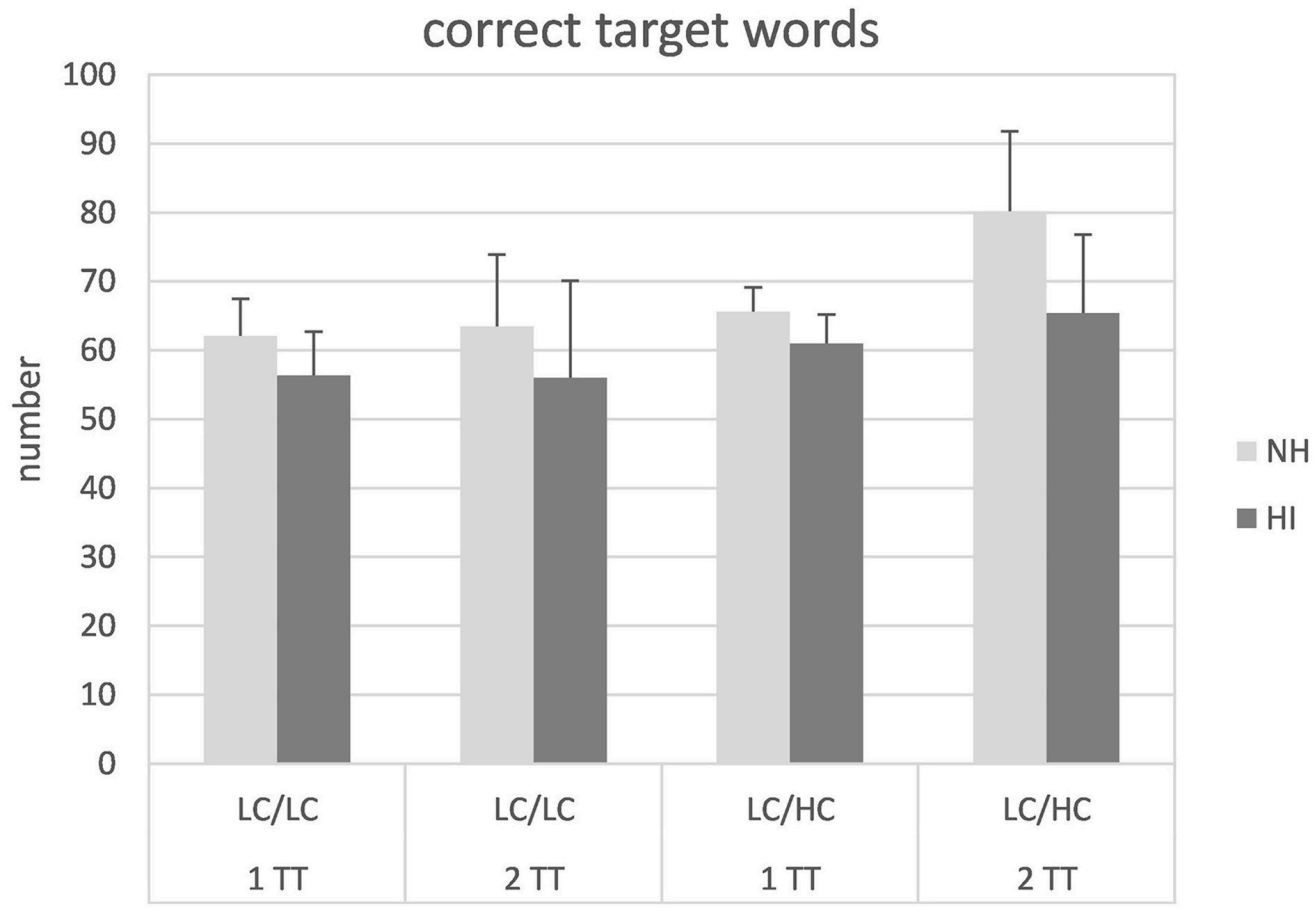
**Number of correctly repeated back target words in normal-hearing (NH) and hearing-impaired (HI) listeners for the different tasks and stimulus types**. For abbreviations see Figure 1.

Figure 2 shows the number of target words repeated back correctly. In general, the average number of correct target words was higher in the NH compared to the HI listeners and also appeared to be higher for LC/HC condition compared to the LC/LC condition. Subjecting the data to a mixed design ANOVA revealed significant main effects of stimulus type [*F*_(1, 26)_ = 38.24, *p* < 0.001] and group [*F*_(1, 26)_ = 9.15, *p* = 0.006]. Moreover, a significant interaction stimulus type × task [*F*_(1, 26)_ = 10.29, *p* = 0.004] could be observed. *Post-hoc* independent samples *t*-tests revealed that the 1TT and the 2TT tasks revealed similar outcome in the LC/LC condition but that in the LC/HC condition more target words were repeated back correctly in the 2TT than in the 1TT task [*t*_(1, 27)_ = 4.25, *p* < 0.001]. No other main effects or interactions were significant.

Three different error types, namely substitutions, confusions and omissions were documented. Subjecting the number of errors to a mixed model ANOVA with error type as the within- and group as the between-subjects variable revealed a significant main effect of error type [*F*_(2, 220)_ = 202.75, *p* < 0.001] and a significant interaction error type × group [*F*_(2, 220)_ = 3.46, *p* = 0.033]. *Post-hoc t*-tests showed that omissions occurred more frequently than substitutions and confusions [*t*_(1, 111)_ = 18.61, *p* < 0.001, *t*_(1, 111)_ = 15.7 *p* < 0.001], and that the listener two groups showed a significant difference for substitutions [*t*_(1, 111)_ = 3,05, *p* = 0.03], a strong trend toward significance for omissions [*t*_(1, 111)_ = 1,86, *p* = 0.065] but no significant differences in confusions (*p* < 0.05). No other main effects or interactions were significant.

**Figure 3:**
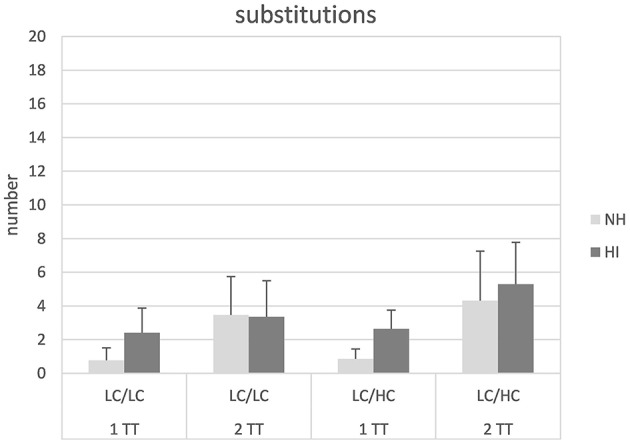
**Number of substitutions in normal-hearing (NH) and hearing-impaired (HI) listeners for the different tasks and stimulus types**. For abbreviations see Figure 1.

Figure 3 shows the number of substitutions (i.e., misunderstood words) for the different conditions and groups. In general, substitutions appeared to be more numerous for the HI than the NH listeners and for the 2TT compared to the 1TT task. Subjecting the data to a mixed design ANOVA revealed significant main effects of task [*F*_(1, 26)_ = 49.24, *p* < 0.001], stimulus type [*F*_(1, 26)_ = 5.42, *p* = 0.028], and group [*F*_(1, 26)_ = 6.28, *p* = 0.02]. Moreover, a significant interaction task × group [*F*_(1, 26)_ = 9.93, *p* = 0.004] could be observed. *Post-hoc* independent samples *t*-tests revealed that the group-difference in substitutions was only significant in the 1TT condition [*t*_(1, 54)_ = 5.99, *p* < 0.001]. No other main effects or interactions were significant.

**Figure 4:**
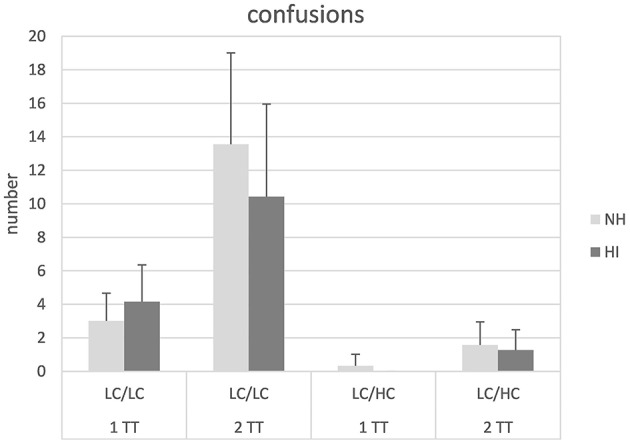
**Number of confusions in normal-hearing (NH) and hearing-impaired (HI) listeners for the different tasks and stimulus types**. For abbreviations see Figure 1.

The number of confusions (i.e., mixing up the male and the female talker) for the different conditions is shown in Figure 4. Apparently, confusions were higher for the 2TT task than for the 1TT task and also higher for low context speech compared to high context speech. Subjecting the data to a mixed design ANOVA revealed significant main effects of task [*F*_(1, 26)_ = 71.49, *p* < 0.001] and stimulus type [*F*_(1, 26)_ = 264.64, *p* < 0.001]. Furthermore, a significant interaction of task × stimulus type [*F*_(1, 26)_ = 5.07, *p* = 0.033] was found. *Post-hoc* paired comparison *t*-tests confirmed that the difference in confusions between the 1TT and the 2TT task was significantly larger for the LC/LC condition compared to the LC/HC condition [*t*_(1, 27)_ = 2.15, *p* = 0.04]. No other main effects or interactions were significant.

**Figure 5:**
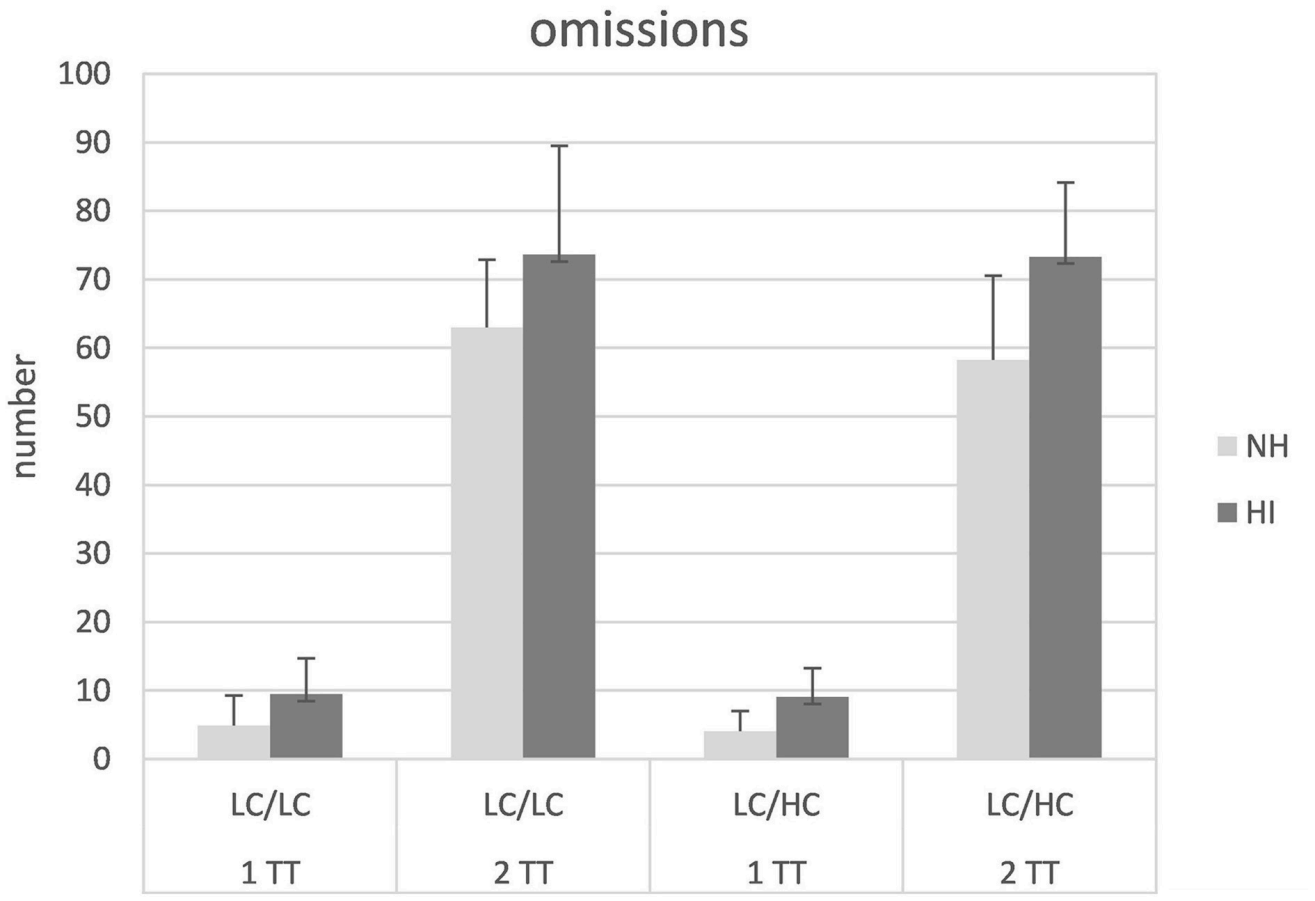
**Number of omissions in normal-hearing (NH) and hearing-impaired (HI) listeners for the different tasks and stimulus types**. For abbreviations see Figure 1.

Figure 5 shows the number of omissions (i.e., not repeating words back) in the different conditions. Compared to substitutions and confusions, omissions occurred clearly more frequently. Subjecting the data to a mixed design ANOVA revealed significant main effects of task [*F*_(1, 26)_ = 871.57, *p* < 0.001], and group [*F*_(1, 26)_ = 13.98, *p* = 0.001]. Furthermore, a significant interaction task × group could be observed [*F*_(1, 26)_ = 9.91, *p* = 0.004]. *Post-hoc t*-tests revealed that the difference between the 1TT and the 2TT task was significantly larger in the HI than the NH listeners [*t*_(1, 54)_ = 3,7, *p* = 0.001]. No other main effects or interactions were significant.

**Figure 6:**
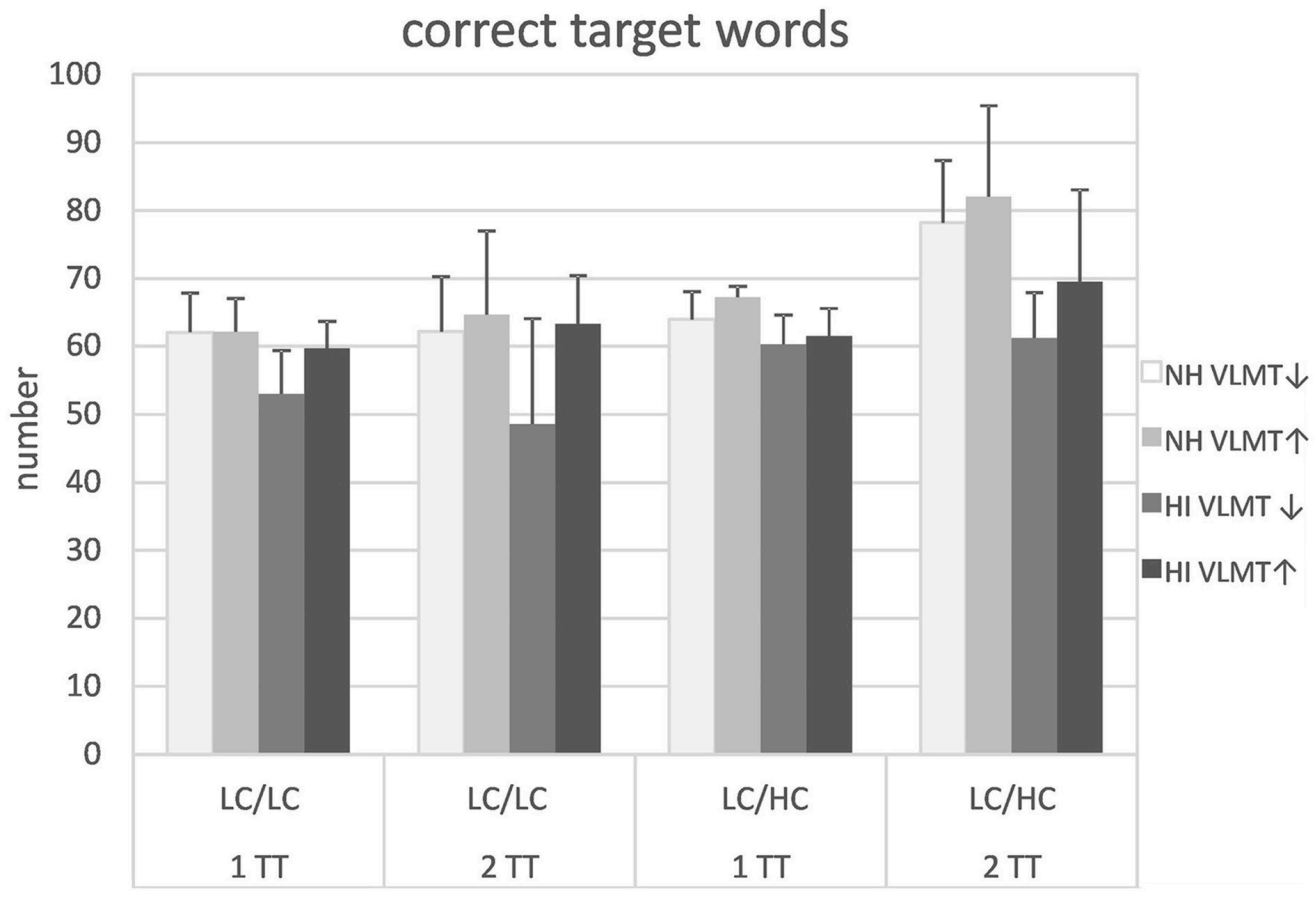
**Target word recognition in normal-hearing (NH) and hearing-impaired (HI) listeners for the different the different tasks and stimulus types**. Listeners were split into groups with respect to their performance in the verbal learning and memory test (VLMT↑, VLMT↓). For abbreviations see Figure 1.

Following the significant group difference for omissions and the model assumptions regarding the importance of WMC for speech recognition in adverse conditions, the participants were further characterized with respect to their VLMT scores in a *post-hoc* analysis. Figure 6 shows the target word recognition of the NH and HI listeners, each subdivided into groups with above-median (VLMT↑) and below-median (VLMT↓) scores. Importantly, this characterization yielded a similar hearing loss for the VLMT↑ and the VLMT↓ participants in the HI listeners. Furthermore, below-median performers in the NH and HI group did not show significantly different VLMT scores and the same held for the above-median performers (see Table 2). It appeared that the VLMT-split did not largely affect target word recognition in the NH listeners, whereas it had a greater effect on the results of the HI listeners. Subjecting the data to a mixed design ANOVA revealed significant main effects of stimulus type [*F*_(1, 24)_ = 44.63, *p* < 0.001], group [*F*_(1, 24)_ = 10.84, *p* = 0.003], and VLMT score [*F*_(1, 24)_ = 4.84, *p* = 0.038]. The significant main effects of stimulus type and group reflect the outcome already presented in Figure 2. Additionally, a significant main effect could be observed for the VLMT-based separation, with those participants with higher scores revealing better target word recognition. Furthermore, as with the data presented in Figure 2 there was a significant stimulus type × task interaction [*F*_(1, 24)_ = 9.68, *p* = 0.05]. Additionally, a significant stimulus type × group × VLMT interaction [*F*_(1, 24)_ = 4.59, *p* = 0.042] could be observed, suggesting greater differences in target word recognition between the two VLMT-groups in the HI listeners than in the NH listeners, especially for low context speech. *Post-hoc t*-tests revealed that only the VLMT-group difference in the HI listeners with the LC/LC condition was significant [*t*_(1, 26)_ = 2,70, *p* = 0.012]. No other main effects or interactions were significant.

## Discussion

Several studies have addressed speech recognition with competing talkers and mainly focused on differences between younger and elderly listeners. These examinations demonstrated that older listeners performed worse than younger listeners even when group differences in hearing loss were taken into account (Humes et al., 2006) and that difficulties already occur in middle-aged persons (Helfer and Freyman, 2014). The present study examined the effects of hearing impairment and two attention tasks differing in cognitive load on speech recognition with competing talkers. The theoretical framework comprised the mechanisms relevant to auditory scene analysis and the interplay of speech and memory aspects as proposed by the ELU model. Two groups of older adults with and without hearing loss were closely matched with regard to age and their neuropsychological profile. We hypothesized that the hearing impaired participants would experience difficulties on different stages of speech processing, namely object formation, stream segregation, and word recall and that the difficulties were especially pronounced in the task with higher cognitive load.

Analysis of the *overall* number of words repeated back revealed that both task and group showed a significant effect. The former could simply be explained with the fact that in the 2TT task more words might be repeated back *per se* than in the 1TT task. However, it was noticeable that the hearing impaired listeners were in average not able to repeat back more than about 70 words per list in the 2TT task which corresponds to the maximum number of words that might be repeated back in the 1TT task. Indeed, there was a significant task × group interaction revealing that the task-difference was significantly smaller in the HI than the NH listeners who showed low performance especially in the more demanding 2TT task. It should be noted that the two groups were closely matched with respect to their neuropsychological profile including a measure of WMC. Thus, the group difference in the overall number of words repeated back cannot simply be attributed to group differences in recall abilities.

Analysis of the number of *correctly* repeated back target words revealed significant main effects of stimulus type and group and a significant interaction of task × stimulus type. The beneficial effect of context with the LC/HC stimulus type was significant in the more demanding 2TT task. In general, the NH listeners were able to correctly repeat back a higher number of target words than the HI listeners but both groups benefitted from context in a similar manner. We are not aware about studies focusing on competing talkers that address the use of context information in persons with and without hearing loss. However, our finding that the normal hearing and the hearing impaired listeners benefitted similarly from context is in line with findings of Benichov et al. (2012), who assessed final-word recognition in sentences masked with noise (i.e., the so called closure paradigm). Different levels of context information were given with the sentences with higher context facilitating better final-word recognition. Benichov et al. examined three different groups (“good hearing”, slight-mild hearing loss with averaged pure-tone hearing loss (PTA) of 16–40 dB HL and moderate hearing loss with 41–60 dB HL). They found that the group with moderate hearing loss benefitted most from context information whereas the listeners with good hearing and slight-mild hearing loss revealed similar benefit. In our HI listeners, PTA ranged from 22 to 49 dB HL thus predominantly representing slight-mild hearing loss.

### Error types

Different errors limited the ability to repeated back target words correctly. In line with the theoretical framework we specified three different error types, namely substitutions, confusions, and omissions. Analysis revealed a significant difference in the occurrence of these errors and a significant error type × group interaction.

Substitutions were due to misunderstanding words and hearing loss resulted in a significantly larger number of substitutions. This is not surprising since the hearing loss considered here typically causes misperception of high-frequency speech sounds, possibly resulting in misunderstanding words. With regard to the theoretical considerations outlined in the introduction, it might be suggested that substitution errors are associated with failures in word object formation. The process underlying word object formation is argued to be a remapping of the speech signal from one encoding acoustic attributes to one representing its phonemic components (Steinschneider et al., 2014). Hearing loss results in impaired acoustic encoding, especially with respect to temporal fine structure, which is in turn relevant for speech understanding in adverse conditions (Anderson et al., 2013). Consequently, failures in object formation were greater in the HI listeners than in the NH listeners. As already discussed in Meister et al. (2013), there were also significant main effects of stimulus type and task. The latter reflects a higher number of substitutions in the 2TT compared to the 1TT task. This can be interpreted as an effect demonstrating that cognitive load might impair the accuracy of acoustic encoding and thus auditory acuity (Rönnberg et al., 2008). Recently, Mattys and Palmer (2015) have shown that the participants' discrimination of phonemes in a divided attention task (auditory plus visual stimulation) decreased, since they tended to select more similar sounding stimuli under higher cognitive load. This is in line with the present study, with substitutions predominantly stemming from similar sounding, yet different words (such as “Dosen” vs. “Rosen”). This increase in substitutions with higher cognitive load was less pronounced in the HI group (see significant task × group interaction), who revealed more substitutions in general. Presumably, the stronger impact of sensory impairment might have toned down the effect of cognitive load on substitutions, as observed in the NH listeners.

Confusion errors might be associated with failures in stream segregation. They depended on the task and the stimulus type, but not on the study group. More confusions were found with the 2TT than with the 1TT task and with low context speech than with high context speech. Both voice characteristics associated with the different “gender” of the talker as well as linguistic characteristics (LC/LC vs. LC/HC) seemed to be beneficial for auditory stream segregation, and these cues largely remained useful for the participants with age-related hearing loss. There is evidence that sensorineural hearing loss is associated with worsened processing of temporal fine structure cues that might deteriorate F0 discrimination (Moore and Glasberg, 2011) and might thus affect stream segregation and speech recognition with competing talkers of different gender (Lee and Humes, 2012). On the other hand, recent physiological data suggested that models exclusively based on temporal fine structure and/or envelope cues do not fully account for the discrimination thresholds assessed in behavioral tests (Kale et al., 2014). Thus, other factors such as central processing noise might also play an important role. Whatever the exact mechanisms in F0 discrimination are, the differences in acoustic cues between the two voices obviously provided robust talker information. Given the relatively low number of confusions this largely facilitated stream segregation in both NH and HI listeners. In our stimuli, additionally formant frequencies of the utterances differed by about 16%, though this cue might be less effective, as Mackersie et al. (2011) have shown that hearing-impaired subjects are restricted in the use of formant frequency changes. Furthermore, linguistic properties also seemed to provide useful information for stream segregation, especially helpful with the more demanding 2TT task (see significant task × stimulus type interaction). Our sentences revealed regular syntax but differed with respect to semantic properties (i.e., low context vs. high context). An examination of syntactical effects on speech recognition with competing talkers was recently described by Kidd et al. (2014). Similar to our methods they used two competing talkers differing in voice cues (and/or location). These were defined as “low-level” cues promoting segregation of speech streams. Sentences had either regular syntax (i.e., name, verb, numeral, adjective, object) or were a random variation of the five-word structure. Syntax was considered to be a “high-level” cue relying on top-down processes and a priory language knowledge. Results obtained in young normal hearing listeners revealed that both, low-level and high-level cues served to select a specific talker and to maintain the focus of attention. Syntax even showed a beneficial effect on target word recall when no low level cues were available. As with our differences in semantic properties of the sentences it was suggested that better predictability due to regular syntax and high context aids performance in competing talker conditions.

The most frequently observed error type was omissions, and there were significant main effects of task and group. Predictably, more omissions occurred with the more demanding 2TT task that required to repeat back words from two target talkers instead of only one target talker. Furthermore, the hearing-impaired participants revealed consistently more omissions than the normal-hearing listeners though the groups were carefully matched with regard to their WMC. It should be noted that this observation also seems not to be due to the slight-moderate hearing loss *per se*, since speech recognition was near perfect when the sentences were presented at 70 dB SPL without competing masker. Thus, it is unlikely that HL rendered single words completely unintelligible. It might be speculated that the increased amount of omissions in the HI group additionally reflects increased cognitive load due to the sensory impairment and the corresponding mechanisms proposed by the ELU model: The hearing loss of the participants might have disrupted the rapid and automatic matching of the entries in the episodic buffer and representations in LTM. As a consequence of the implicit process disruption, a compensatory mechanism taxing WMC might be invoked—labeled “explicit processing” by Rönnberg (2003) and Rönnberg et al. (2008, 2013). Together with the finding that the representation of words in short-term memory seems to be less stable with hearing impairment than with normal hearing (Pichora-Fuller et al., 1995), this might explain the larger proportion of omissions observed in the HI listeners compared to the NH participants. The significant task × group interaction suggests that the 2TT task was especially detrimental to the HI listeners. Thus, under increased cognitive load there might be extra difficulties for hearing impaired listeners due to the combined effects of hearing loss and the higher attentional and memory requirements.

### *Post-hoc* group splitting

Following the results discussed above a *post-hoc* group splitting with respect to WMC was performed. Though this additional separation resulted in a relatively low number of observations, a further significant main effect of VLMT score as well as a further significant interaction of VLMT score, listener group, and stimulus type could be shown for target word recognition. The main effect of VLMT score revealed that participants with higher WMC repeated back more target words than those with lower WMC. It could be argued that this simply reflects similarities between the VLMT paradigm and the speech recognition tests as both require to repeat back words. However, the significant interaction of VLMT score, listener group, and stimulus type revealed that the effect of group-split regarding the VLMT score held only for the HI listeners in the LC/LC condition. This suggests that WMC was especially important for the HI listeners when they could not rely on context information. In the LC/LC condition the HI listeners with better WMC approached the results of the NH listeners. This might be interpreted in the sense of a compensatory effect of cognitive function on the detrimental impact of hearing loss. However, due to the small group size in the *post-hoc* analysis this finding should be treated with caution and requires a more comprehensive examination. Nevertheless, it seems in line with Wingfield and Stine-Morrow (2000) and Wingfield et al. (2015) who showed that contextual cues might dilute some of the effects of limited WMC.

Taken together, the results suggest an interplay of hearing loss and cognitive load regarding speech recognition with competing talkers. We anticipated that the hearing impaired listeners would experience difficulties on different stages of speech processing. This held true for word object formation and word recall, but not for stream segregation. We also hypothesized that difficulties for the HI listeners would be pronounced in the more demanding 2TT compared to the 1TT attention task. The results suggest extra difficulties with higher cognitive load for the HI compared to the NH listeners as they were especially limited in repeating back words in the 2TT task, though both groups showed near perfect speech recognition in quiet and showed similar outcome in their neuropsychological profile. When the groups were further separated with respect to their WMC it appeared that the HI listeners could use working memory in the sense of a compensatory mechanism with regard to the detrimental effects of hearing loss—especially when no supporting context cues were available. However, due to the relatively small number of participants this has to be examined further. Apart from these differences found between the groups the results revealed that context could be used by the normal hearing listeners and the listeners with typical age-related hearing loss in a similar manner.

## Author contributions

HM designed the work, analyzed the data and wrote the manuscript, SS acquired the data and revised the manuscript critically, MO, SR, and MW revised the manuscript critically.

### Conflict of interest statement

The authors declare that the research was conducted in the absence of any commercial or financial relationships that could be construed as a potential conflict of interest.
